# Bayesian hierarchical model for transcriptional module discovery by jointly modeling gene expression and ChIP-chip data

**DOI:** 10.1186/1471-2105-8-283

**Published:** 2007-08-03

**Authors:** Xiangdong Liu, Walter J Jessen, Siva Sivaganesan, Bruce J Aronow, Mario Medvedovic

**Affiliations:** 1 Department of Environmental Health, University of Cincinnati, 3223 Eden Ave. ML 56, Cincinnati, Ohio 45267, USA; 2 Division of Biomedical Informatics, Cincinnati Children's Hospital Medical Center, Cincinnati, Ohio 45229, USA; 3 Mathematical Sciences Department, University of Cincinnati, Cincinnati, OH 45221, USA

## Abstract

**Background:**

Transcriptional modules (TM) consist of groups of co-regulated genes and transcription factors (TF) regulating their expression. Two high-throughput (HT) experimental technologies, gene expression microarrays and Chromatin Immuno-Precipitation on Chip (ChIP-chip), are capable of producing data informative about expression regulatory mechanism on a genome scale. The optimal approach to joint modeling of data generated by these two complementary biological assays, with the goal of identifying and characterizing TMs, is an important open problem in computational biomedicine.

**Results:**

We developed and validated a novel probabilistic model and related computational procedure for identifying TMs by jointly modeling gene expression and ChIP-chip binding data. We demonstrate an improved functional coherence of the TMs produced by the new method when compared to either analyzing expression or ChIP-chip data separately or to alternative approaches for joint analysis. We also demonstrate the ability of the new algorithm to identify novel regulatory relationships not revealed by ChIP-chip data alone. The new computational procedure can be used in more or less the same way as one would use simple hierarchical clustering without performing any special transformation of data prior to the analysis. The R and C-source code for implementing our algorithm is incorporated within the R package *gimmR *which is freely available at http://eh3.uc.edu/gimm.

**Conclusion:**

Our results indicate that, whenever available, ChIP-chip and expression data should be analyzed within the unified probabilistic modeling framework, which will likely result in improved clusters of co-regulated genes and improved ability to detect meaningful regulatory relationships. Given the good statistical properties and the ease of use, the new computational procedure offers a worthy new tool for reconstructing transcriptional regulatory networks.

## Background

Transcriptional regula tion is one of the crucial mechanisms used by living systems to maintain homeostasis. Disregulation of gene expression underlies toxic effects of many chemicals [1], and gene expression changes are often reliable markers of a disease [2]. The specificity of transcriptional initiation of a eukaryotic gene is maintained through a complex interaction of one or more sequence-specific transcription factors, regulatory DNA regions harboring corresponding DNA regulatory motifs, chromatin-remodeling proteins and the basal transcriptional machinery [3]. While not all modes of expression regulatory controls are known, it has been shown that in many important biological processes the initiation of transcription requires binding of one or more transcriptional factors to their cognate regulatory motifs within regulatory DNA regions. Two key high-throughput (HT) experimental technologies are capable of producing data offering insights into the expression regulatory mechanism on a genome scale. The first technology are expression microarrays facilitating simultaneous monitoring expression of virtually all genes in a genome [3-5]. The second technology is the Chromatin Immuno-Precipitation on Chip (ChIP-chip) technology facilitating assessment of transcription factor binding events on a genomic scale [6,7]. Optimal joint modeling of data generated by these two complementary biological assays, with the goal of identifying and characterizing TMs, is an important open problem in computational biomedicine.

Earliest applications of microarray technology included attempts at discovering shared regulatory motifs and corresponding transcription factors within groups of co-expressed genes identified by cluster analysis [8]. Groups of co-expressed genes were first identified by clustering gene expression profiles. Putative regulatory motifs inducing the co-expression were then identified de-novo using the MEME algorithm [9]. The inefficiency of procedures in which different data-types (e.g. expression data and promoter sequences) are analyzed separately is due to the inability of patterns in different data-types to re-enforce each other. For example, due to the noise in microarray data, the correlation between expression levels of two co-regulated genes could be too weak to be detected by clustering expression data alone. However, if evidence exists that promoters of these two genes are bound by the same TF, this information could enforce the weak signal in the expression data and allow us to identify these two genes as being parts of the same TM. In the traditional two-step approach such co-regulation will be lost since the second step regulatory motif analysis is conditional on co-expression of the two genes.

Several heuristic algorithms have been developed for constructing TMs by integrated analysis of gene expression and binding (ChIP-chip) data. Genetic Regulatory Modules (GRAM) algorithm [10] uses binding data to identify a gene set bound to common TFs (p-value < 0.001). It then searches for other genes at a lower level of significance (p-value < 0.01) that are bound by those TFs and have similar expression levels to the initial gene set core (d < d_0_). ReMoDiscovery [11] follows similar stringent and relaxed two step procedures and infers TMs from Chip-chip, motif and expression data. Module Finding Algorithm (MOFA) also uses two level p-values, but additional criteria for selecting genes regulated by a specific TF is the correlation between expression levels of such genes and expression level of the TF [12]. Statistical-Algorithmic Method for Bicluster Analysis (SAMBA) algorithm [13] transforms expression and binding data items to properties of genes/genes or genes/proteins, then generates a genes-properties bipartite graph. The algorithm aims at discovering sets of genes with statistically significant common properties. SAMBA requires discretization of inherently continuous gene expression and binding data based on more or less ad-hoc cut-offs which will almost certainly reduce the information content of the data.

In a model-based approach to find TMs based on gene expression and TF binding data, one postulates the probabilistic model of all data and then estimates parameters of the model which define TM membership. Three such models based on Bayesian networks have been proposed. In the first approach [14] both gene expression and ChIP-chip data are directly modeled within the same Bayesian hierarchical model. In the other model, ChIP-chip data is used to calculate prior probabilities of TM memberships [15] based on an extension of the Bayesian module networks model [16]. In both of these models, the number of the modules has to be first be estimated from the data (or guessed) and all inference is valid conditional on the number of modules being correct. Since both of these models can also be thought of as extensions of the basic finite-mixture model, it is very likely that they will share inherent instability with respect to misspecification of the "correct" number of modules [17,18]. Earlier, a Bayesian casual network inferred from discretized expression data was used to describe the gene regulatory network with the binding data used to establish the constraints for the network structure [19]. The number of genes participating in the network construction is limited because of the complexity of model search. COGRIM [20] algorithm uses a Bayesian hierarchical framework to fit a gene-by-gene linear regression model of a gene's expression levels as function of is a quadratic function of all TFs' expression levels and their pair-wise interactions. The ChIP-chip binding data and the TF binding motif scores based on predefined Position Weight Matrices (PWM) are integrated as the prior information in the model. Genes are grouped into same TMs if they are regulated by the same set of TFs.

We developed a novel Expression-ChIP Infinite Mixture (ECIM) model for identifying TMs by jointly modeling gene expression and TF binding data. The model is constructed by extending the context-specific infinite mixture model (CSIMM) [21] in such a way that expression and binding data are represented by two separate contexts with different probabilistic models. We also constructed a novel probabilistic representation for the ChIP-chip data that seems to capture all relevant information from this data and use it within the binding-context of the model. The overall approach makes use of the Bayesian infinite mixture framework [17,18] to circumvent the issue of identifying the 'correct' number of global and local patterns in the data. Context-specificity not only allows the use of different probabilistic models to represent expression and binding data, but it also allows for discordances between patterns of co-expression and co-regulation. Posterior distribution of model parameters is estimated using Gibbs sampling [22]. TMs are formed based on Posterior Pairwise Probabilities (PPPs) of co-membership and Posterior Binding Probabilities (PBPs). It has been previously shown that PPPs can be directly interpreted as measures of statistical significance of co-membership [18,21].

The new computational procedure can be used in more or less the same way as one would use simple hierarchical clustering without need to perform any special transformation of data prior to the analysis. In the results section we show that PBPs are able to identify binding relationships not revealed by CHIP-chip binding data alone. We demonstrate the ability of this procedure to integrate information from gene expression and TF binding data by assessing the functional coherence of TMs constructed from real-world datasets.

## Results and Discussion

### Data preparation

We constructed four expression-binding datasets to examine the performance of ECIM and alternative methods. For each dataset, binding data consisted of ChIP-Chip data assessing binding affinities for 106 TFs to promoters of 6270 genes [6]. Expression datasets we used were the sporulation data set consisting of gene expression measurements throughout the sporulation process for the yeast strain SK1 [8]; the sporulation data set consisting of gene expression measurements during sporulation for the yeast strains SK1 and W303y [23]; the cell cycle data set consisting of gene expression measurements spanning two complete yeast cell cycles [24]; and the combined sporulation-cell cycle dataset which we previously used to validate the CSIMM model [21]. Dual channel data [8] was processed by: (i) adjusting for background signal intensities; (ii) calculating log-intensity ratios of intensities in two channels; (iii) adjusting log-ratios using local regression of log-ratios on average log-intensities in two channels; and (iv) centering each gene's log-ratios by subtracting the gene-specific average log-ratio. Affymetrix data [23,24] was processed by: (i) setting any measurement below one to one; (ii) log-transforming measurements; and (iii) centering each gene's log-measurements by subtracting the gene-specific average log-measurement. Genes with the maximum signal strength of less than 100 were excluded from the analysis. To make results comparable across different datasets, we used only data for genes represented on all microarray platforms (4980 genes).

### Sensitivity and specificity of co-memembership in TMs

Using the Gibbs sampler, we generated a sequence of TMs approximating the marginal posterior distribution of TMs given data. This distribution was summarized by calculating PPPs of two genes belonging to the same TM, and PBP of a specific TF binding to the promoter of a specific gene. For each dataset we constructed an Expression-ChIP Infinite Mixture (ECIM) based hierarchical clustering of genes using PPPs as the similarity measure with the average-linkage principle. The precision of such analysis was compared to results obtained by using alternative analytical approaches and by using the equivalent models with only expression or only binding data. Following are descriptions of all methods compared:

#### ECIM Expression and Binding

Hierarchical clustering based on PPPs derived from ECIM analysis of both expression and binding data.

#### ECIM Expression

Hierarchical clustering based on PPPs derived from ECIM analysis of expression data.

#### ECIM Binding

Hierarchical clustering based on PPPs derived from ECIM analysis of binding data.

#### Binding P-Values

TM's formed based on p-values of binding calculated in the original publication[6].

#### Binding PBP

TMs formed based on PBPs from ECIM analysis of expression and binding data.

#### Euclidian Distance

Hierarchical clustering based on Euclidian distances of expression data.

#### GRAM

TMs formed using the GRAM algorithm with default parameters, expression and binding data.

#### SAMBA

TMs formed using the SAMBA algorithm with default parameters and expression data only.

ROC curves were constructed by correlating results for the 949 KEGG-associated genes where "functional clusters" are based on the co-membership of these 949 genes within any KEGG [25] pathway. It is obvious that this is not the perfect "gold standard" as some co-regulated genes will not be categorized to belong to a common pathway and vice versa. However, the assumption behind using membership in specific pathways as a gold standard, which is that co-regulated genes are more likely to participate in the same pathway than randomly grouped genes, is reasonable. Other well-known annotation databases, such as GO [26] or MIPS [27], are more complicated to use since they are hierarchically structured and results would depend on the level of specificity used to construct functional grouping.

#### ROC for hierarchical TMs based on hierarchical clustering using PPPs and Euclidian distance

The tree was cut at different depths to create clustering with every possible number of clusters. For a fixed number of clusters a pair of genes (from the 949 genes assigned to at least one pathway) belonging to the same cluster was assumed to be a "true positive" if the two genes both belonged to at least one specific KEGG pathway, and it was considered to be a "false positive" if they did not share a single KEGG pathway. True and false positive rates were then obtained by dividing the number of true/false positives with the total number of gene pairs sharing a common KEGG pathway and total number of gene pairs not sharing a KEGG pathway respectively. When the number of clusters is equal to the number of genes and all genes are placed in their own individual clusters, both true and false positive rates are equal to zero. A ROC curve is defined when we reduce the number of clusters and both true and false positive rates increase. At the extreme when all genes are placed in the same cluster, both true and false positive rates are equal to one.

#### ROC curves for ECIMs based on binding p-values and PBP

The significance cut-off was varied between 0 and 1. For each cut-off level, two genes were considered to be co-regulated if they were bound by at least one common TF at this significance level. True and false positive rates were established in the same way as for the clusters formed by hierarchical clustering with KEGG "gold standard".

#### GRAM and SAMBA

True and false positive rates for TMs produced by the two algorithms with default parameters were calculated in the same way as for the previous two situations. There was no recommended way to vary specificity and sensitivity of these two algorithms so we report only a single true and false positive rate for each algorithm.

**Figure 1:**
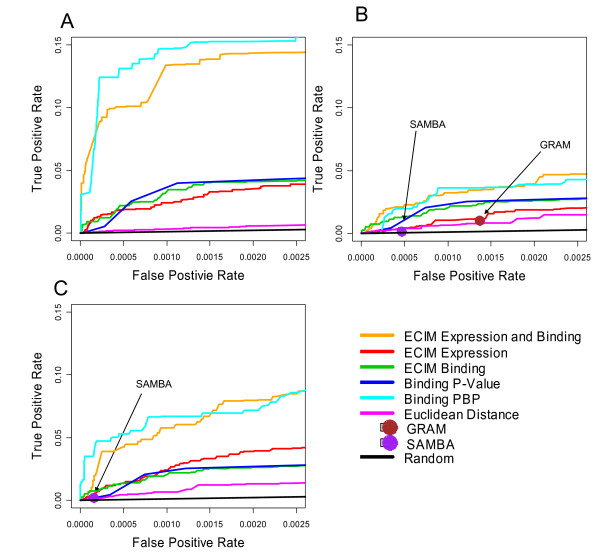
ROC curves for 8 different algorithms using three different yeast gene expression datasets. A) Chu,1998, sporulation; B) Primig,2000, sporulation;C) Cho,1998, cellCycle and the ChIP-chip data of Lee, 2002. KEGG pathways were used as the gold standard. ECIM utilizing both expression and binding data dominated all other algorithms. ROC "spots" for GRAM and SAMBA algorithms were obtained by applying the algorithms using the default parameters.

**Figure 2:**
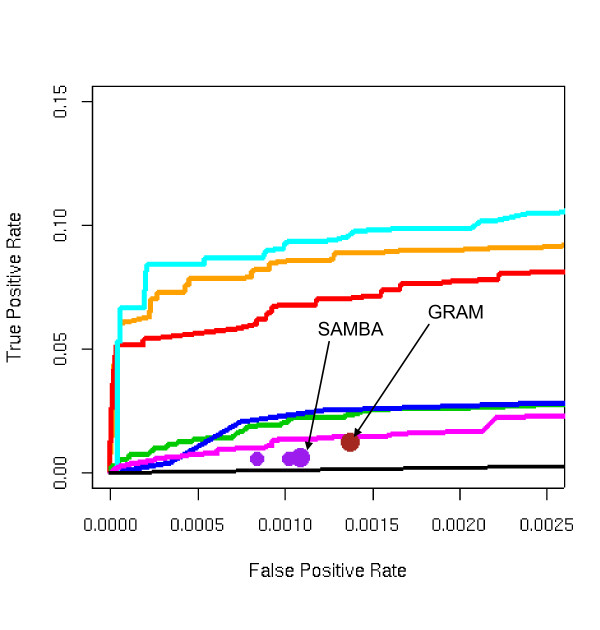
ROC curves for 8 different algorithms using the combined sporulation and cell-cycle gene expression dataset and the ChIP-chip data of Lee, 2002. KEGG pathways were used as the gold standard. ECIM utilizing both expression and binding data dominated all other algorithms. ROC "spots" for GRAM and SAMBA algorithms were obtained by applying the algorithms using the default parameters. Smaller ROC "spots" for SAMBA were obtained by systematically manipulating algorithm's parameters.

Since just 5% of gene-pairs annotated in KEGG shared the same pathway, only when the True Positive Rate (TPR) is at least 20 times higher than the False Positive Rate (FPR) do true positive pairs outnumber the false positives. Therefore we only show ROC curves for each dataset/method combination for statistically relevant false-positive rates (less than 0.05). The FPRs achieved by GRAM and SAMBA are around or less than 0.001, thus we plotted left most part of ROC curves (less than 0.0025) to make a clear comparison (Figure 1). ROC curves on the expended rage of FPRs (less than 0.05) are shown Figure 2.

ECIM-derived TMs based on the expression and binding data clearly outperformed all other approaches. In all three datasets, ECIM framework was able to successfully integrate information from both data types and significantly improve precision of analysis over individually analyzing any one of two data types. When using only binding data, it made no difference whether we simply use p-values to construct modules or apply ECIM procedure using only the binding data context, which was expected since the binding data was the only information source even we use different processing methods. On the other hand, TMs constructed by either hierarchically clustering genes using PPPs or using PBPs derived from the same analysis, were equally precise. This suggests that either PPP or PBP summarizes the posterior distribution of TMs generated by the ECIM analysis of two data types and carries all the meaningful information about the underlying TM structure.

**Figure 3:**
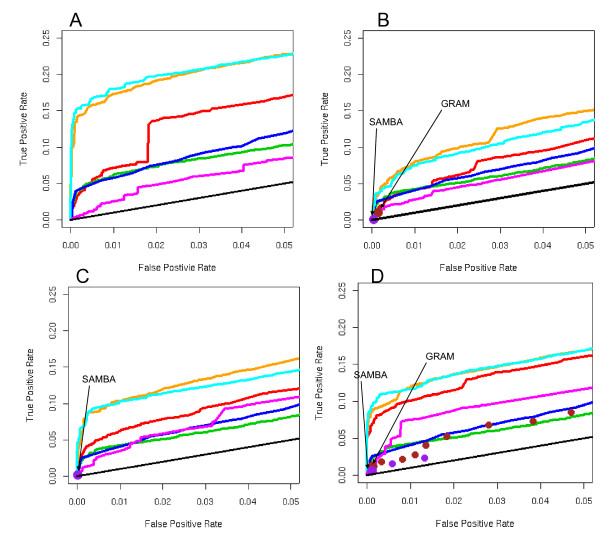
ROC curves from Figures 1 and 2 with expanded range for FPR. ROC curves for 8 different algorithms using four different yeast gene expression datasets. A) Chu,1998, sporulation; B) Primig,2000, sporulation;C) Cho,1998, cellCycle, D) combined sporulation and cell-cycle dataset Liu,2006, and the ChIP-chip data of Lee, 2002. KEGG pathways were used as the gold standard. ECIM utilizing both expression and binding data dominated all other algorithms. Large ROC "spots" for GRAM and SAMBA algorithms were obtained by applying the algorithms using the default parameters. Smaller ROC "spots" for GRAM and SAMBA were obtained by systematically manipulating algorithm's parameters.

To demonstrate the seamless integration of ECIM framework with more sophisticated expression data models we re-analyzed the combined sporulation-cellcycle data set we previously described [21] using CSIMM model for multiple expression data contexts (Figure 2 and Figure 3D). As expected, the ECIM with CSIMM expression data contexts outperformed all other approaches, indicating the ability of the CSIMM model to effectively integrate information from different expression data sets and the ability of the ECIM model to integrate further such complex expression data with ChIP-Chip binding data.

The performance of two previously described computational procedures for constructing TMs based on joint analysis of expression and binding data was relatively poor. Points defined by single pairs of true/false positive rates for both methods with default parameters fall below all ROC curves including the one that uses only binding p-values. For the combined sporulation-cellcycle dataset we manipulated the parameters for the two algorithm with the goal of obtaining ROC points for a range of false positive rates. Detailed tables of parameters used and resulting FPRs and TPRs are shown in Supplemental Tables 1 and 2, (see Additional files 2 and 3 ). ROC points obtained by these two algorithms with non-default parameters are depicted by smaller dots in Figures 2 and 3D. While we managed to expand the range of FPRs, the overall conclusions did not change.

In the case of SAMBA we used only expression data because we could not establish with certainty the appropriate transformation for the binding data used in the original study [13]. This is appropriate because the statistical procedure implemented in SAMBA is same for both the gene expression and appropriately transformed ChIP-chip data. Furthermore, SAMBA has been originally described in the context of clustering gene expression data alone and the web page manual describes only this kind of use. However, it is important to emphasize that SAMBA's performance should be compared to results of other procedures that use only gene expression data (Euclidian Distance and ECIM Expression). Given the poor precision of TMs generated by SAMBA when compared to ECIM using only expression data, we conjecture that adding binding data is unlikely to improve SAMBA's results to the point of performing better than ECIM using both data types. For the sporulation data in Figure 1A both SAMBA and GRAM failed to identify any TMs. Same was the case for GRAM with cell-cycle data in Figure 1C.

In the original publications, both SAMBA and GRAM were used to analyze larger expression datasets than we used here. To assess the scalability of results presented here we also analyzed a significantly larger dataset with 165 microarray experiments assessing yeast transcriptional responses to various environmental perturbations [28]. The functional coherence of produced TMs was also compared to the functional coherence of TMs previously constructed using a large scale gene expression data analysis [29] for 23 different cut levels provided by authors, and two latest algorithms (ReMoDiscovery and COGRIM) [11,20] for constructing TMs from jointly analyzing gene expression data, ChIP-chip data and DNA motif scores obtained by scanning gene promoters using predefined PWM. The comparisons to ReMoDiscovery and COGRIM were based on results published in original publications describing these two algorithms. These results were based on analyzing the gene expression datasets that contained the Gasch dataset [28], and on the same TF binding dataset we used in our analyses (Lee's ChIP-chip data [6]). We downloaded module definitions from the respective support web sites and constructed ROC points using again KEGG pathways as the gold standard. For ReMoDiscovery we used two modules definitions discussed in the paper (seed module and extended module). For COGRIM we used two modules defined by authors (B+C+ corresponding to modules defined by COGRIM and supported by binding data alone and B-C+ corresponding to modules defined by COGRIM but not supported by binding data alone) and the combined module corresponding to all modules constructed by COGRIM. Unfortunately, after multiple attempts we were not able to construct TMs using SAMBA on this dataset. This could be a consequence of the large number of missing values in this dataset or our inability to correctly format missing values. We again manipulated GRAM parameters (details in Supplemental Table 1, (see Additional file 2) to expand the range of false positive rates.

**Figure 4:**
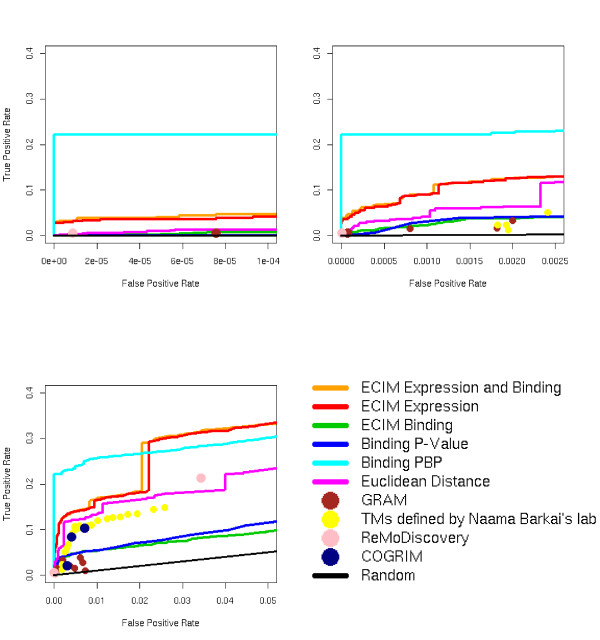
ROC curves for 7 different algorithms using the combined dataset of yeast transcriptional responses to various environmental changes [28] and the ChIP-chip data of Lee, 2002. We used the experiments with at least 5 time points or dose-response points for the total of 165 microarrays. KEGG pathways were used as the gold standard. The functional coherence of produced TMs was also compared to the functional coherence of TMs constructed by three other algorithms utilizing expression datasets containing the Gasch dataset used in our calculations. ROC points for TMs constructed by Naama Barkai's lab [11] utilizing only a very large expression dataset at 23 different cut-off levels are depicted by yellow spots. The seed module and extended module constructed by ReMoDiscovery [11] utilizing gene expression, ChIP-chip and binding sequence motif data are depicted by pink spots. B+C+, B-C+ and the C+ TMs constructed by combining B+C+ and B-C+ modules identified by COGRIM [20] utilizing gene expression, ChIP-chip and binding sequence motif data are depicted by dark blue spots. All three diagrams represent the same ROC curves/plots for different ranges of False Positive Rates (x-axis) ECIM results again dominated all other algorithms in terms of functional coherence.

Basic conclusion still held and all algorithms we tested produced improved ROC results when compared to the smaller expression datasets (Figure 4). However, although ECIM performed as well or better than any other algorithm, significant improvements in precision from adding ChIP-chip data were visible only when PBP's are used to construct the modules. This could be the consequence of the additional noise in the algorithm for constructing hierarchical clustering from PPPs. ECIM also outperformed TMs constructed by the large gene expression datasets alone [26] as well as two algorithms that use expression, binding and DNA sequence motif information to infer TMs [11,20] despite the dramatically smaller number of data points used in the analysis. COGRIM outperformed GRAM and matched the functional coherence of modules that were based on a much larger gene expression dataset alone. This could be due to the additional regulation information used in the analysis or simply due to the more efficient use of the expression data alone.

**Figure 5:**
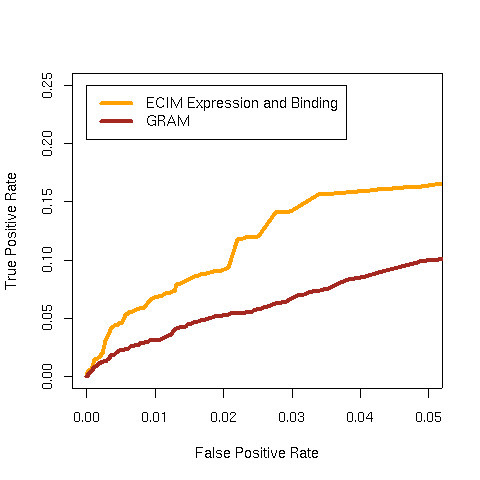
ROC curves comparing the functional coherence of TMs constructed by GRAM and ECIM using the combined sporulation and cell-cycle gene expression dataset and the ChIP-chip data of Lee, 2002 with Gene Ontologies as gold standard.

Finally, we performed additional comparisons between TMs produced by GRAM and ECIM using Gene Ontologies as the gold standard [26]. In this comparison, we constructed TMs by cutting the hierarchical tree constructed by the ECIM algorithm so that the total number of genes in resulting TMs was about the same as the number of genes implicated by GRAM (740 unique genes in 98 TMs). For each gene-pair we identify the most specific GO category to which both of them belong by defining the specificity as *I *= *[1-log2(S/2)/log2(N/2)] *where *S *is the number of genes annotated in this GO item and *N *is the total number of genes annotated in GO. It has been shown that such a measure of specificity is a good way to represent the level of information about functional relationship between genes based on GO groupings [30]. For a specific cut-off *i*, a pair of genes is True Positive if the corresponding *I>i *and are placed in at least one common TM. A pair of genes is False Positive if *I>i*, but the two genes do not share a commong TM. ROC curves in Figure 5 are constructed by systematically changing the threshold *i *and calculating corresponding true and false positive rates for TMs constructed by GRAM and those constructed by ECIM. Results of this analysis are concordant with results obtained by using KEGG pathways as the gold standard.

**Figure 6:**
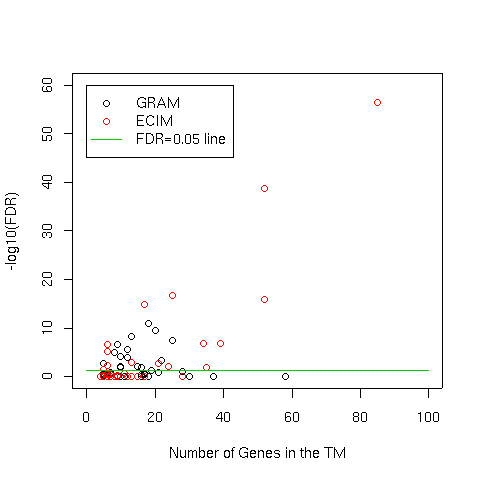
The distribution of TM sizes vs -log_10 _of FDR-adjusted p-values calculated by Fisher's test for association between the membership in a TM and the most significantly over-represented Gene Ontology. The green line represents the statistically significant cut-off of FDR<0.05. All points above the line represent statistically significant associations.

In addition to constructing ROC curves we examined the coherence of TMs identified in this analysis in terms of statistical significances of over-represented Gene Ontologies. For each TM, we identified the most over-represented Gene Ontology as measured by the p-value of the Fisher's exact test. The distribution of TM sizes and the statistical significances of most over-represented Gene Ontologies is depicted in Figure 6. Assuming that the false discovery rate of 0.05 to be statistically significant, the results of the analysis are summarized in Table 1. Overall, the higher proportion of TMs constructed by ECIM (15 out of 51 vs 15 out of 94) were statistically significantly associated with at least on Gene Ontology. The number of genes in statistically significant TMs constructed by ECIM was more than twice the number of genes in statistically significant TMs constructed by GRAM.

**Table 1:** Summaries of associations between TMs and Gene Ontologies

	Number of Genes Assigned to TMs	Number of TMs	Number of TMs Significantly Correlated With At Least One GO (Fisher's FDR<0.05)	Number of Genes in Significant TMs
GRAM	740	94	15	206
ECIM	740	51	15	425

The comparison of Gene Ontologies significantly associated with TMs constructed by ECIM and GRAM (Table 2) reveals that several key Gene Ontologies were implicated by both algorithms (protein biosynthesis, Sporulation, sulfur metabolism, mitosis and amino acid metabolism). On the other hand, 8 out of 15 ECIM modules and 5 out 15 GRAM modules were algorithm specific. All of these 13 algorithm specific categories could be linked in one way or another to the two basic process investigated by expression data (sporulation and cell cycle). Consequently, it seems that both algorithms are identifying relevant TMs, it is just that ECIM is assigning a greater number relevant genes to these TMs. The list of all TMs along with the associated Gene Ontologies is given in the Supplemental Table 3, (see Additional file 4).

**Table 2:** Functional comparison of TMs constructed by ECIM and GRAM. Several key Gene Ontologies were implicated by both algorithms (bold text with matching numbers).

**FDR adjusted p-value**	**GO categories associated with ECIM modules**	**GO categories associated with GRAM modules**	**FDR adjusted p-value**
2.7E-57	**protein biosynthesis (1)**	oxidative phosphorylation	9.9E-12
2.1E-39	ribosome biogenesis and assembly	**amino acid metabolism (5)**	2.9E-10
1.6E-17	meiosis	**protein biosynthesis (1)**	4.7E-09
1.6E-16	**spore wall assembly (sensu Fungi) (2)**	**Sporulation (2)**	3.7E-08
1.4E-15	**sulfur metabolism (3)**	**sulfur metabolism (3)**	2.3E-07
1.2E-07	**spore wall assembly (sensu Fungi) (2)**	**protein biosynthesis (1)**	3.3E-06
1.8E-07	DNA replication	**protein biosynthesis (1)**	8.8E-06
2.7E-07	arginine biosynthesis	Glycolysis	6.8E-05
5.7E-06	ribosome biogenesis	**protein biosynthesis (1)**	1.3E-04
1.5E-03	**mitosis (4)**	de novo' IMP biosynthesis	6.1E-04
2.4E-03	lagging strand elongation	chromatin assembly or disassembly	1.6E-03
4.7E-03	**amino acid biosynthesis (5)**	**sulfur metabolism (3)**	6.8E-03
1.0E-02	**mitotic cell cycle (4)**	alcohol catabolism	7.3E-03
1.6E-02	cytokinesis, completion of separation	**interphase of mitotic cell cycle (4)**	1.2E-02
3.6E-02	protein neddylation	**mitotic sister chromatid cohesion (4)**	1.4E-02

### Constructing TM's and identification of associated regulators

**Figure 7:**
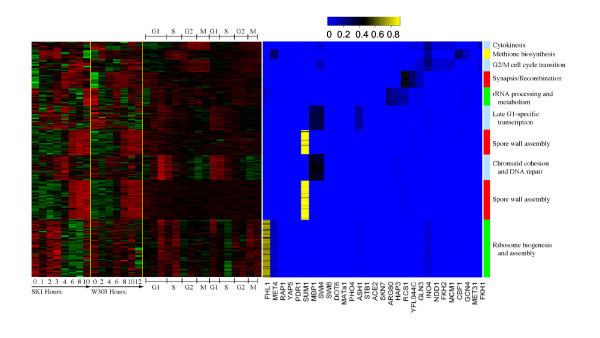
Heatmap of expression data and PBPs for highly specific TMs inferred by ECIM algorithm using the combined sporulation and cell-cycle gene expression dataset and Lee's ChIP-chip data. Each line in the heatmap represents a gene. Red-green heatmap represents gene expression levels in the three different gene expression datasets that were combined together in this analysis and each column represents one microarray. The yellow-blue heatmap represents Posterior Binding Probabilities for 29 most significant TFs with each column in the heatmap representing a TF. Colour-bar on the right of the heatmap depicts groupings of co-regulated genes into TMs and is denoted with the significantly correlated functional category.

**Figure 8:**
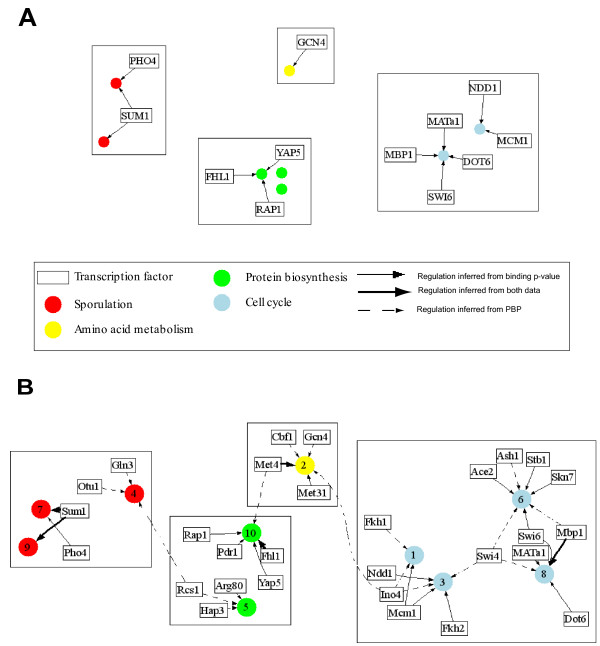
Transcriptional regulatory network based on TMs associated with 4 key biological processes implicated by the analysis, also depicted in Figure 7. A) TMs constructed with expression data only. B) TMs constructed using expression and binding data

To demonstrate the simplicity of use and interpretation of ECIM results we constructed TMs based on results of the combined sporulation-cell cycle dataset. 294 genes were selected based on the fact that their average linkage distance based on ECIM-derived PPPs to at least one other gene or group of genes was below 0.1 and their cluster size is larger than 10. Previously we demonstrated that such average linkage distance cut-offs have direct interpretations in terms of statistical significance of implicated associations [21]. The heatmap in Figure 7 depicts clusters of co-regulated genes and their associated TFs as well as the strength of this association based on PBPs. On the right hand side of the heat-map, are gene ontologies most significantly associated with each TM. All TFs associated with different TM's (Figure 8) are identified based on either the high PBPs between individual genes and individual TFs, or by over-representation of genes with statistically significant binding p-values (<0.001) for a TF in the ChIP-chip experiment (see methods). Descriptions of resulting TMs are given in the Supplementary Table 4, (see Additional file 5). The biological meaning of identified TMs is discussed in the next section.

We also investigate the utility of PBPs in identifying novel regulatory relationships not implicated by ChIP-chip data alone. We used experimentally verified binding site lists from TRANSFAC [31] consisting of 174 binding sites involving 127 genes and 57 TFs as our gold-standard. Among all gene/TF pairs with binding p-values less than 0.001, 35% are verified in TRANSFAC. The threshold 0.001 was carefully selected to balance the specificity and sensitivity of binding between all TFs and gene promoters [6]. We selected 8 gene/TF pairs (around 0.1% of the total) with highest PBPs among all 7239 (127*57) pairs in TRANSFAC. None of the 8 pairs had binding p-values less than 0.001. However, 3 of the 8 gene/TF pairs (SIP4/SIP4, CLN2/SWI4, SMK1/SUM1) are listed in TRANSFAC. The accuracy rate (0.37) is almost same as binding p-value's, which suggests that PBP is able to identify novel regulation information. Another two gene/protein pairs (SPR3/SUM1, OPI3/INO4) are very good candidates for further investigation. SPR3's promoter region has SUM1's putative binding site MSE and its transcription is increased with the presence of SUM1 [32], OPI3's promoter region has INO4's putative binding site UASINO element, its transcription is depressed with the presence of INO4[33,34].

### Description of transcriptional modules detected

#### Sporulation

Clusters associated with the biological processes of synapsis/recombination and spore wall assembly were clearly discerned in the Primig sporulation dataset (Figure 7). Genes within each of the clusters for both yeast strains SK1 and W303 were all upregulated late in the sporulation process. Joint data clustering showed enrichment in the number of clusters associated with sporulation as well as the number of regulators identified (Figure 8). In addition to modules regulated by Sum1 and Pho4, the ECIM algorithm identified a third transcriptional module associated with synapsis/recombination (cluster 4) that consisted of three additional regulators; Gln3, Otu1 and Rcs1. Gln3 positively regulates genes that are subject to nitrogen catabolite repression (NCR)[35]; under conditions of nitrogen limitation, Gln3 localizes to the nucleus and activates NCR-sensitive genes. Gln3 was likely detected due to the use of nitrogen-deficient sporulation media. In addition to its role as a deubiquitylation enzyme, Otu1 has been suggested by database mining to affect *PIS1 *expression, which is required for the final step in phosphatidylinositol synthesis[36]. Previous work has demonstrated that *S. cerevisiae *inositol auxotrophic strains require inositol for the completion of sporulation[37]. Rcs1 is a transcription factor involved in iron utilization and homeostasis [38]. Previous studies have found that it is also involved in controlling cell size [39] as well as biotin uptake and biosynthesis, nitrogen assimilation and purine biosynthesis[40]. Using joint data clustering, two transcriptional modules separately detected Sum1. *SUM1 *is required for middle sporulation element-mediated repression during meiotic development in *S. cerevisiae *[32].

#### Amino acid metabolism

A single transcriptional module involved in the biological process of amino acid metabolism was detected using expression data exclusively. This ten gene Gcn4-regulated module could not be further specifically annotated. In contrast, joint data clustering identified a transcriptional module that was significantly associated with methionine biosynthesis (cluster 2 in Figure 8). Genes in this module were cell cycle regulated and had increased expression in the S/G2 transition (Figure 7). This "MET" cluster has similarly been observed using microarrays to study *S. cerevisiae *cell cycle-regulated genes[41]. In addition to Gcn4, the primary regulator of the transcriptional response to amino acid starvation, joint data clustering identified Met4, Met31, Cbf1 and Ino4 (Figure 8). Met4 is responsible for the regulation of the sulfur amino acid pathway and requires different combinations of auxiliary factors including Met31 and Cbf1. In the Primig sporulation dataset [23], genes in the cluster associated with methionine biosynthesis show a derepression early in the sporulation process prior to sporulation clusters associated with spore wall assembly. Ino4 is required for derepression of inositol-choline-regulated genes involved in phospholipid synthesis. Previous work has shown that the completion of sporulation requires inositol [37].

#### Protein biosynthesis

Three clusters associated with the biological processes of rRNA processing and metabolism, RNA processing and ribosomal gene expression, and mitochondrial ribosomal protein metabolism were detected using expression data exclusively. However, only one transcriptional module was identified, consisting of the regulators Fhl1, Rap1 and Yap5. This same cluster was identified using joint data clustering (cluster 10) and two additional regulators were identified; Met4 and Pdr1. Patterns identified in both sporulation and cell cycle datasets suggested that genes regulated by this module were upregulated in G1- and S-phases and/or early in SK1 sporulation. The forkhead-like transcription factor Fhl1 plays a key role in the control of rRNA processing [42]. Rap1, in its role as a positive regulator, activates a number of ribosomal proteins [43]. Yap5 is a bZIP protein, shown to be regulated at the G1/S transition [44]. Pdr1 is a master drug regulator involved in the recruitment of other zinc cluster proteins to pleiotropic drug resistance elements to modulate the regulation of multidrug resistance genes [45]. Met4, also identified above in the amino acid metabolism transcriptional module category, is a transcription factor involved in the regulation of the sulfur amino acid pathway.

The second transcriptional module involved in the biological process of rRNA processing and metabolism (cluster 5) was identified using joint data clustering and consisted of three additional regulators; Arg80, Hap3 and Rcs1. Patterns identified in the Cho cell cycle dataset [24] suggested that genes regulated by this module were upregulated in S-and G2-phases. The ReMoDiscovery algorithm similarly identified Arg80 associated with ribosome biogenesis [11], a transcription factor involved in regulation of arginine-responsive genes. Likewise, the GRAM algorithm identified Rcs1 associated with protein synthesis [10]. Rcs1, also identified above in the sporulation transcriptional module category, is a transcription factor involved in a variety of different processes, including iron homeostasis, control of cell size, biotin biosynthesis, nitrogen assimilation and purine biosynthesis. Hap3 is a subunit of the CCAAT-binding factor (CBF), which activates genes required for respiratory metabolism; the Hap2 and Hap3 subunits of CBF are also required for optimal expression of ASN1, an asparagine synthase [46].

#### Cell cycle

Two transcriptional modules involved in the biological processes of chromatin cohesion and DNA repair and G2/M cell cycle transition were detected using expression data exclusively. Joint data clustering also identified these two modules (clusters 3 and 8), but found several more regulators. In addition to Dot6, MATa1, Mbp1, Mcm1, Ndd1 and Swi6, the CSIMM algorithm identified Fkh2, Ino4 and Swi4. Further, two additional transcriptional modules associated with the biological processes of late-G1-specific transcription (cluster 6) and cytokinesis (cluster 1) were detected (Figure 8) and included the regulators Ace2, Ash1, Mbp1, Skn7, Stb1 and Swi4 as well as Fkh1, Ino4 and Mcm1.

In diploid cells, MATa1 has been shown to interact with another homeodomain protein, MATalpha2, and bind DNA as a heterodimer to repress transcription of haploid-specific genes [47]. Mbp1 is a DNA-binding protein that forms the MBF complex with Swi6; MBF is a sequence-specific transcription factor that regulates gene expression during the G1/S transition of the cell cycle [48]. In addition to Mbp1, Swi6 has been shown to form the SBF complex with the DNA-binding protein Swi4 to regulate transcription at the G1/S transition [49]. The MBF and SBF complexes regulate late-G1-specific transcription. Although Skn7 is required for induction of heat-shock genes by oxidative stress [50], it has recently been shown to associate with Mbp1, forming a transcription factor independent of MBF that may be involved in the bud-emergence process [51]. Stb1 binds to Swi6 and has a role in the regulation of MBP-specific transcription [52]. Mcm1 has been shown to be required for the coordination of G2-specific transcription [53]. Ndd1 is essential for the expression of a set of late-S-phase-specific genes [54]. Fkh1 and Fkh2 are transcription factors of the forkhead family that regulate the cell cycle [55]. Ace2 has been shown to activate the expression of early-G1-specific genes [56]. Dot6 is a protein of unknown function involved in telomeric silencing [57] and filamentation [58]. Ino4, also identified above using joint expression and binding data clustering in the amino acid metabolism transcriptional module category, is a transcription factor that regulated genes involved in phospholipid synthesis. In diploid cells deprived of nitrogen, Ash1 has been shown to be asymmetrically localized to the nuclei of daughter cells during pseudohyphal growth [59].

Finally, the transcriptional coherence of the genes in these TMs and associated regulators were assessed by calculating average correlations between expression levels of genes in a TM and the expression levels of associated TFs (Table 3). The statistical significance of these average correlations (*r*) was assessed by calculating p-values based on resampling-based null-distribution of average correlations. Briefly, for each TM-TF pair a random set of genes of the same size as the original TM was selected from the list of all genes used in the analysis. The average correlation between the expression levels of the TF and all genes in such random set was calculated and compared to the actual average correlation for this TM-TF pair. This was repeated 2000 times. For r>0, one-sided p-value assessing the statistical significance of r was calculated as the proportion of times when *r *was larger than re-sampled average correlations. For r<0, one-sided p-value was calculated as the proportion of times when *r *was smaller than re-sampled average correlations. Two-sided p-values were obtained by doubling the one-sided p-values and are reported in Table 3. P-values that were equal to zero by this calculations were set to the smallest observable non-zero p-value (0.001). 23 out of 37 TM-TF pairs were significantly positively or negatively correlated (p-value< 0.05). Expected number of pairs with p-value<0.05 under the global null hypothesis that none of the TM-TF pairs were correlated is less than 2. 15 out of 23 TFs were positively correlated with respective TMs representing putative inducers. 8 TM-TF pairs were negatively correlated implicated potential repressors.

**Table 3:** Average correlations between expression levels of genes in a TM and the expression levels of associated TFs.

Factor	Module	Module description	Correlation with module	P value of correlation
FKH1	1	Cell Cycle	0.53	0.001
MCM1	1	Cell Cycle	0.14	0.007
INO4	1	Cell Cycle	0.22	0.029
GCN4	2	Amio Acid Metabolism	0.09	0.539
CBF1	2	Amio Acid Metabolism	0.17	0.079
MET4	2	Amio Acid Metabolism	0.00	0.497
INO4	2	Amio Acid Metabolism	0.33	0.001
MET31	2	Amio Acid Metabolism	-0.14	0.789
SWI4	3	Cell Cycle	0.01	0.689
MCM1	3	Cell Cycle	0.04	0.079
FKH2	3	Cell Cycle	-0.24	0.001
INO4	3	Cell Cycle	0.48	0.001
NDD1	3	Cell Cycle	0.39	0.001
GLN3	4	Sporulation	-0.21	0.001
YFL044C	4	Sporulation	0.09	0.6
RCS1	4	Sporulation	-0.37	0.001
HAP3	5	Protein Biosynthesis	0.12	0.003
RCS1	5	Protein Biosynthesis	-0.24	0.001
ARG80	5	Protein Biosynthesis	0.09	0.142
MBP1	6	Cell Cycle	-0.02	0.858
SWI4	6	Cell Cycle	0.02	0.841
SKN7	6	Cell Cycle	-0.12	0.882
ASH1	6	Cell Cycle	0.24	0.156
ACE2	6	Cell Cycle	-0.13	0.003
SWI6	6	Cell Cycle	0.12	0.227
STB1	6	Cell Cycle	0.15	0.001
SUM1	7	Sporulation	0.23	0.001
PHO4	7	Sporulation	0.39	0.001
MBP1	8	Cell Cycle	-0.29	0.001
DOT6	8	Cell Cycle	0.16	0.001
SWI4	8	Cell Cycle	0.32	0.001
SWI6	8	Cell Cycle	0.03	0.445
SUM1	9	Sporulation	0.26	0.001
PDR1	10	Protein Biosynthesis	-0.18	0.001
MET4	10	Protein Biosynthesis	0.06	0.001
RAP1	10	Protein Biosynthesis	-0.17	0.001
FHL1	10	Protein Biosynthesis	0.04	0.001

In this paper we utilized the ChIP-chip dataset of Lee et al [6] instead of the newer ChIP-chip dataset[60]. The reason for this was the "higher information density" in the Lee dataset which has about 4000 statistically significant binding events while the newer dataset has about 25% more binding events for twice as many transcription factors examined. However, we did perform similar analysis using the newer dataset for comparative reasons. ROC curves resulting from this analysis (Figure S1 in the web supplement, (see Additional file 1)) and TMs (Supplementary Table 5, (see Additional file 6) were similar to the ones discussed here.

## Conclusion

We presented a novel probabilistic model and related computational procedures for jointly modeling the gene expression and TF binding data within the context specific Bayesian infinite mixture framework. The algorithm identifies transcriptional modules consisting of groups of co-regulated genes and TFs that regulate expression of genes within such groups. The method does not require prior knowledge of number of modules. We demonstrated the improved functional coherence of TMs by analyzing real world data. We also demonstrated that novel regulatory relationship can be identified which would not be implicated by either analyzing gene expression or binding data separately. The new method also produced more functionally coherent TMs than two alternative algorithms for joint analysis of gene expression and binding data. In the original publications, both of these algorithms were tested on much larger expression datasets than we used here. However, the functional coherence as measured by the sensitivity and specificity of predicting the co-membership in KEGG pathways remained significantly improved for the ECIM algorithm in analyzing an order of magnitude larger dataset [28]. Furthermore, most of the expression datasets examining a specific biological process are similar in size to datasets we used here and so the comparisons we made are very relevant.

Since there are no free parameters to adjust or tune during clustering phase, users only need to provide the data and the time consuming sampling process will go by itself, then user can select or change either stringent or relaxed criteria to search qualified gene group and corresponding TFs immediately. The output will show results of the analysis in a familiar form without the need to completely understand the mathematical/computational machinery used. We believe that this is an appealing characteristic of ECIM. The model presented here does not account for combinatorial interactions of different TFs in regulating expression. However, the modular nature of the model allows straightforward incorporation of more precise models for ChIP-chip data which will most likely further improve the performance of the method.

## Methods

### The probabilistic model and computational algorithm

Suppose that expression levels are measured for *T *genes across *M *experimental conditions. If *x*_*im *_is the expression level of gene *i *for experimental condition *m*, then ***x***_*i *_= (*x*_*i1*_, *x*_*i2*_, ..., *x*_*iM*_) denotes the complete expression profile for gene *i*. Suppose further the ChIP-Chip experiments measured binding affinity of *N *TFs to promoters of each of *T *genes. If *p*_*ij *_is the p-value for rejecting the null-hypothesis that TF *j *does not bind the promoter of gene *i*, we define the "binding intensity" of TF *j *to promoter of gene *i *as *y*_*ij *_= log(*p*_*ij*_)/log(*p*_min_), where *p*_*min *_is the minimum of all p-values. ***y***_*i *_= (*y*_*i1*_, *y*_*i2*_, ..., *y*_*iN*_) denotes the complete "binding profile" for gene *i*. ***x***_*i *_and ***y***_*i *_jointly represent the expression-binding (EB) *profile *for gene *i*.

Each gene's EB profile can be viewed as being generated by one out of *Q *different underlying EB *patterns*. Suppose that *c*_*i *_is the classification variable indicating the EB pattern that generates EB profile *i. c*_*i *_*= q *means that EB profile *i *was generated by pattern *q*. A clustering structure indicating putative TMs is defined by a set of classification variables for all EB profiles ***C ***= (*c*_*1*_, *c*_*2*_, ..., *c*_*T*_). The expression part of pattern *q *that generates profile *i *is represented by the mean vector and the variance-covariance matrix of the M-dimensional Gaussian random variable (*μ*_*q*_, **Σ**_*q*_). The binding part of pattern *q *is N-dimensional vector ***b***_*q *_= (*b*_*q1*_, ..., *b*_*qN*_), where *b*_*qj *_∈ {0,1} and ∑j=1Nbqj=1
 MathType@MTEF@5@5@+=feaafiart1ev1aaatCvAUfKttLearuWrP9MDH5MBPbIqV92AaeXatLxBI9gBaebbnrfifHhDYfgasaacH8akY=wiFfYdH8Gipec8Eeeu0xXdbba9frFj0=OqFfea0dXdd9vqai=hGuQ8kuc9pgc9s8qqaq=dirpe0xb9q8qiLsFr0=vr0=vr0dc8meaabaqaciaacaGaaeqabaqabeGadaaakeaadaaeWbqaaiabdkgaInaaBaaaleaacqWGXbqCcqWGQbGAaeqaaaqaaiabdQgaQjabg2da9iabigdaXaqaaiabd6eaobqdcqGHris5aOGaeyypa0JaeGymaedaaa@399C@, specifying the identity of the TF binding to promoters of genes in TM *q *(*b*_*qj *_= 1 implicates that TF *j *is associated with genes in TM *q*). The space of all possible associated TFs is augmented by a "baseline" TF having p-value of 0.5 for all genes. This allows certain expression patterns not to be associated with any real TF.

Observed expression profiles of genes from the same TM (i.e. generated by the same expression pattern) are assumed to be a random sample from the same multivariate Gaussian random variable (e.g. *c*_*i *_*= q *implies that ***x***_*i *_~*N*_*M*_(*μ*_*q*_, Σ_*q*_)). The binding profiles of genes associated with TM *q*, {***y***_*i *_: *c*_*i *_= *q*}, are assumed to be observations from the random variable with probability density function defined as

p(yi)=∏j=1Np(yij)
 MathType@MTEF@5@5@+=feaafiart1ev1aaatCvAUfKttLearuWrP9MDH5MBPbIqV92AaeXatLxBI9gBaebbnrfifHhDYfgasaacH8akY=wiFfYdH8Gipec8Eeeu0xXdbba9frFj0=OqFfea0dXdd9vqai=hGuQ8kuc9pgc9s8qqaq=dirpe0xb9q8qiLsFr0=vr0=vr0dc8meaabaqaciaacaGaaeqabaqabeGadaaakeaacqWGWbaCcqGGOaakieWacqWF5bqEdaWgaaWcbaGaemyAaKgabeaakiabcMcaPiabg2da9maarahabaGaemiCaaNaeiikaGIaemyEaK3aaSbaaSqaaiabdMgaPjabdQgaQbqabaaabaGaemOAaOMaeyypa0JaeGymaedabaGaemOta4eaniabg+GivdGccqGGPaqkaaa@4203@ where *p*(*y*_*ij*_) = 2(*y*_*ij*_) *if b*_*qj *_= 1 and *p*(*y*_*ij*_) = 2(1 - *y*_*ij*_) *if b*_*qj *_= 0.

The local structure of the expression and binding patterns is specified by the Q × 2 matrix ***L***(***C****) = (****L***_*1*_, ..., ***L***_*Q*_), where *L*_*q1 *_= *k*_*1 *_if genes in TM *q *are placed in group *k*_*1 *_within the expression context and *L*_*q2 *_*= k*_*2 *_if genes in TM *q *are placed in group *k*_*2 *_within the binding context.

### Specification of the complete model

**Figure 9:**
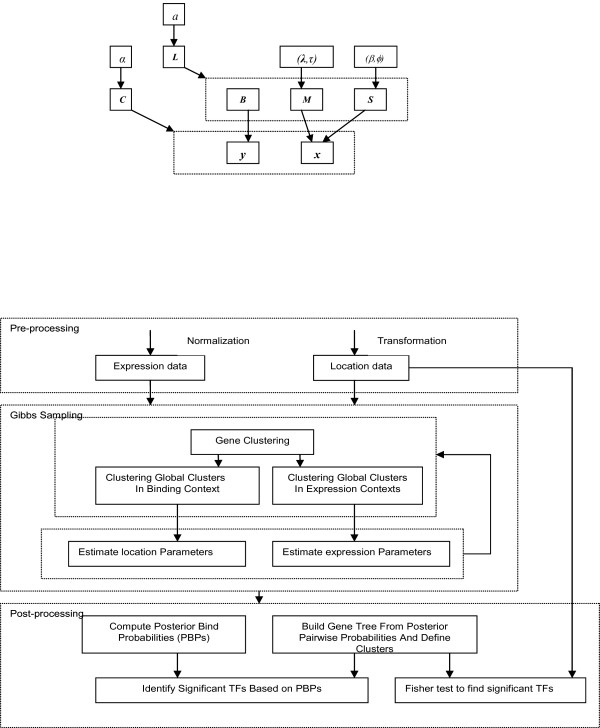
A) The joint probabilistic model for gene expression TF binding data B) The flow chart depicting the complete analysis flow for constructing TMs using ECIM algorithm.

The probabilistic model describing the distribution of the data (i.e. observed EB profiles (***x***_*i*_, ***y***_*i*_)) is given in the form of a Bayesian hierarchical model [61]. Dependencies between various model parameters and the data are defined by the Directed Acyclic Network [62] in Figure 9. Nodes in the network represent random variables and arcs define the independence structure of the joint probability distribution function. An arc drawn between a node and a dotted rectangle containing multiple nodes implies that it is the parent node for all nodes within the rectangle. Assuming that the probability distribution of any node is independent of its non-descendants if values of the parent nodes are given (Directed Markov Assumption), the joint probability distribution of all parameters and data is given by the product of the local probability distributions of individual random variables given their parents.

*p*(***X***, ***Y***, ***C***, ***L***, ***M***, ***S, B***, *α*, *a*, *λ*, *τ*, *β*, *ϕ*) = *p*(***X***|***C***, ***M***, ***S***) *p*(***Y***|***C***, ***B***) *p*(***C***|*α*)*p*(***S***|*β*, *ϕ*) *p*(***L***|***C***, *a*)*p*(***M***|*λ*, *τ*)*p*(*α*)*p*(*a*)*p*(*λ*)*p*(*τ*)*p*(*β*)*p*(*ϕ*),

where ***M ***= {*μ*_*1*_, ..., *μ*_*Q*_} and ***S ***= {Σ_*1*_, ..., Σ_*Q*_} are the set of all mean vectors and variance-covariance matrices defining expression patterns, and ***B ***= {***b***_*1*_, ..., ***b***_*q*_} is the set of corresponding binding patterns. Due to the context-specificity, not all parameters defining EB patterns are unique. That is, (*μ*_*q*_, *Σ *_*q*_) = (*μ*_*q'*_, *Σ *_*q'*_) whenever *L*_*q1*_*= L*_*q'1*_, and ***b***_*q *_= ***b***_*q*' _whenever *L*_*q2 *_= *L*_*q'2*_.

As specified above, *p*(***x***_*i *_| *c*_*i *_= *q*, ***M***, ***S***) = *f*_*N *_(***x***_*i *_| *μ*_*q*_, Σ_*q*_), where *f*_*N*_(.|*μ*,Σ) is the multivariate Gaussian probability distribution function with mean *μ *and variance-covariance matrix Σ, and *p*(***y***_*i *_| *c*_*i *_= *q*, ***B***) = *f*_*pA*_(***y***_*i *_| ***b***_*q*_), where *f*_*PA*_(.|***b***) is the density function given binding vector ***b ***defined in **Eq 1**.

Prior distributions for the local TM assignments ***C ***and context groupings ***L ***are defined following the infinite mixtures approach that avoids the specification of the "correct" number of groups of local clusters for each context[17,18,21]. The prior distribution for ***C ***is defined by specifying prior probabilities that a complete data vector will be either placed in an already existing TM *q*, p( ci=q|C−i,α)= n−i,qT−1+α
 MathType@MTEF@5@5@+=feaafiart1ev1aaatCvAUfKttLearuWrP9MDH5MBPbIqV92AaeXatLxBI9gBaebbnrfifHhDYfgasaacH8akY=wiFfYdH8Gipec8Eeeu0xXdbba9frFj0=OqFfea0dXdd9vqai=hGuQ8kuc9pgc9s8qqaq=dirpe0xb9q8qiLsFr0=vr0=vr0dc8meaabaqaciaacaGaaeqabaqabeGadaaakeaacqWGWbaCcqGGOaakcqqGGaaicqWGJbWydaWgaaWcbaGaemyAaKgabeaakiabg2da9iabdghaXjabcYha8nXvP5wqSXMqHnxAJn0BKvguHDwzZbqegqvATv2CG4uz3bIuV1wyUbacemGaa83qamaaBaaaleaaiiaacqGFsislcqWGPbqAaeqaaOGaeiilaWccciGae0xSdeMaeiykaKIaeyypa0JaeeiiaaYaaSaaaeaacqWGUbGBdaWgaaWcbaGaeyOeI0IaemyAaKMaeiilaWIaemyCaehabeaaaOqaaiabdsfaujab+jHiTiabigdaXiabgUcaRiab9f7aHbaaaaa@57E8@, or that a new TM is created p( ci≠cj ∀j≠i|C−i,α)= αT−1+α
 MathType@MTEF@5@5@+=feaafiart1ev1aaatCvAUfKttLearuWrP9MDH5MBPbIqV92AaeXatLxBI9gBaebbnrfifHhDYfgasaacH8akY=wiFfYdH8Gipec8Eeeu0xXdbba9frFj0=OqFfea0dXdd9vqai=hGuQ8kuc9pgc9s8qqaq=dirpe0xb9q8qiLsFr0=vr0=vr0dc8meaabaqaciaacaGaaeqabaqabeGadaaakeaacqWGWbaCcqGGOaakcqqGGaaicqWGJbWydaWgaaWcbaGaemyAaKgabeaakiabgcMi5kabdogaJnaaBaaaleaacqWGQbGAaeqaaOGaeeiiaaIaeyiaIiIaemOAaOMaeyiyIKRaemyAaKMaeiiFaWNaem4qam0aaSbaaSqaaGGaaiab=jHiTiabdMgaPbqabaGccqGGSaaliiGacqGFXoqycqGGPaqkcqGH9aqpcqqGGaaidaWcaaqaaiab+f7aHbqaaiabdsfaujab=jHiTiabigdaXiabgUcaRiab+f7aHbaaaaa@4EBA@, where ***C***_-*i *_= (*c*_*1*_, *c*_*2*_, ..., *c*_*i*-*1*_, *c*_*i*+*1*_, ..., *c*_*T*_), *n*_-*i*,*q *_is the number of profiles generated by EB pattern *q *without counting EB profile *i*, and *α *is the hyper-parameter. Similarly, local structure priors are specified by the probability that expression or binding profiles from TM *q *are further grouped together within the corresponding context. The probability of assigning TM *q *to an already existing group of TMs *t *within context *f *(*f *= 1 for the expression context and 2 for the binding context), is p(Lqf=t|a)  ∝ n−qftQ−1+a
 MathType@MTEF@5@5@+=feaafiart1ev1aaatCvAUfKttLearuWrP9MDH5MBPbIqV92AaeXatLxBI9gBaebbnrfifHhDYfgasaacH8akY=wiFfYdH8Gipec8Eeeu0xXdbba9frFj0=OqFfea0dXdd9vqai=hGuQ8kuc9pgc9s8qqaq=dirpe0xb9q8qiLsFr0=vr0=vr0dc8meaabaqaciaacaGaaeqabaqabeGadaaakeaacqWGWbaCcqGGOaakcqWGmbatdaWgaaWcbaGaemyCaeNaemOzaygabeaakiabg2da9iabdsha0jabcYha8jabdggaHjabcMcaPiabbccaGiabbccaGiabg2Hi1kabbccaGmaalaaabaGaemOBa42aaSbaaSqaaGGaaiab=jHiTiabdghaXjabdAgaMjabdsha0bqabaaakeaacqWGrbqucqWFsislcqaIXaqmcqGHRaWkcqWGHbqyaaaaaa@48EF@, where *n*_-*qft *_is the number of TMs currently placed in local grouping *t *within context *f *without counting TM *q *and *a *is the hyper-parameter. The probability of assigning TM *q *to a new local group is p(Lqf≠Lq'f,∀q'≠q|a)∝aQ−1+a
 MathType@MTEF@5@5@+=feaafiart1ev1aaatCvAUfKttLearuWrP9MDH5MBPbIqV92AaeXatLxBI9gBaebbnrfifHhDYfgasaacH8akY=wiFfYdH8Gipec8Eeeu0xXdbba9frFj0=OqFfea0dXdd9vqai=hGuQ8kuc9pgc9s8qqaq=dirpe0xb9q8qiLsFr0=vr0=vr0dc8meaabaqaciaacaGaaeqabaqabeGadaaakeaacqWGWbaCcqGGOaakcqWGmbatdaWgaaWcbaGaemyCaeNaemOzaygabeaakiabgcMi5kabdYeamnaaBaaaleaacqWGXbqCcqGGNaWjcqWGMbGzaeqaaOGaeiilaWIaeyiaIiIaemyCaeNaei4jaCIaeyiyIKRaemyCaeNaeiiFaWNaemyyaeMaeiykaKIaeyyhIu7aaSaaaeaacqWGHbqyaeaacqWGrbquiiaacqWFsislcqaIXaqmcqGHRaWkcqWGHbqyaaaaaa@4C93@. Hyper-parameters *a *and *α *are further modeled and estimated from the data and don't have to be specified in the analysis[21,63]. Conditional distributions for all other parameters in the model given their parent nodes in the DAG are the same as previously described [17,18,21] and are given in the web supplement (see Addtional file 1).

The goal of the analysis is to estimate the posterior distribution of parameters in the model given data *p(****C***,***L***, ***M***, ***S, B****, α, a, λ, τ, β, ϕ | ****X***, ***Y***) in the traditional sense of Bayesian statistical analysis. More specifically, we are interested in the marginal distribution of ***C***, ***L ***and ***B ***given (***X***, ***Y***) obtained by integrating out all other parameters *p*(***C***, ***L***, ***B***|***X***, ***Y***) = ∫*p*(***C***, ***L***, ***M***, ***S***, ***B***, *α*, *a*, *λ*, *τ*, *β*, *ϕ*|***X***, ***Y***)*d*(***M***, ***S***, *α*, *a*, *λ*, *τ*, *β*, *ϕ*|***X***, ***Y***)

### Fitting the model

The joint posterior distribution of all parameters in the model given data is estimated using Gibbs sampler. Gibbs sampler [22] is a general procedure for sampling observations from a multivariate distribution. It proceeds by iteratively drawing observations from complete conditional distributions of all components given the current values of all other components. Under mild condition, the distribution of generated multivariate observations converges to the target multivariate distribution. The Gibbs sampler employed here is derived from previously described algorithms for fitting infinite mixture models.

The posterior probability of placing EB profile *i *into an existing TM *q*, given all other parameters is

p(ci=q|C−i,xi,M,S,yi,B)∝n−i,qT−1+α  fN( xi|μq,Σq)fPA(yi|bq)
 MathType@MTEF@5@5@+=feaafiart1ev1aaatCvAUfKttLearuWrP9MDH5MBPbIqV92AaeXatLxBI9gBaebbnrfifHhDYfgasaacH8akY=wiFfYdH8Gipec8Eeeu0xXdbba9frFj0=OqFfea0dXdd9vqai=hGuQ8kuc9pgc9s8qqaq=dirpe0xb9q8qiLsFr0=vr0=vr0dc8meaabaqaciaacaGaaeqabaqabeGadaaakeaacqWGWbaCcqGGOaakcqWGJbWydaWgaaWcbaGaemyAaKgabeaakiabg2da9iabdghaXjabcYha8Hqadiab=neadnaaBaaaleaaiiaacqGFsislcqWGPbqAaeqaaOGaeiilaWIae8hEaG3aaSbaaSqaaiabdMgaPbqabaGccqGGSaalcqWFnbqtcqGGSaalcqWFtbWucqWFSaalcqWF5bqEdaWgaaWcbaGaemyAaKgabeaakiab=XcaSiab=jeacjabcMcaPiabg2Hi1oaalaaabaGaemOBa42aaSbaaSqaaiabgkHiTiabdMgaPjabcYcaSiabdghaXbqabaaakeaacqWGubavcqGFsislcqaIXaqmcqGHRaWkcqaHXoqyaaGaeeiiaaIaeeiiaaIaemOzay2aaSbaaSqaaiabd6eaobqabaGccqGGOaakcqqGGaaicqWF4baEdaWgaaWcbaGaemyAaKgabeaakiabcYha8HGadiab9X7aTnaaBaaaleaacqWGXbqCaeqaaOGaeiilaWccceGaeW3Odm1aaSbaaSqaaiabdghaXbqabaGccqGGPaqkcqWGMbGzdaWgaaWcbaGaemiuaaLaemyqaeeabeaakiabcIcaOiab=Lha5naaBaaaleaacqWGPbqAaeqaaOGaeiiFaWNae8Nyai2aaSbaaSqaaiabdghaXbqabaGccqGGPaqkaaa@74B3@, and the posterior probability of placing EB profile *i *into new TM is

p( ci≠ci',∀i'≠i|C−i,xi,M,S,yi,B)∝               αT−1+α∫fN(xi|μq,Σq)fPA(yi|bq)p(μq,Σq|λ,τ,β,φ)p(bq)d(μq,Σq,bq)
 MathType@MTEF@5@5@+=feaafiart1ev1aaatCvAUfKttLearuWrP9MDH5MBPbIqV92AaeXatLxBI9gBaebbnrfifHhDYfgasaacH8akY=wiFfYdH8Gipec8Eeeu0xXdbba9frFj0=OqFfea0dXdd9vqai=hGuQ8kuc9pgc9s8qqaq=dirpe0xb9q8qiLsFr0=vr0=vr0dc8meaabaqaciaacaGaaeqabaqabeGadaaakqaabeqaaiabdchaWjabcIcaOiabbccaGiabdogaJnaaBaaaleaacqWGPbqAaeqaaOGaeyiyIKRaem4yam2aaSbaaSqaaiabdMgaPjabcEcaNaqabaGccqqGSaalcqGHaiIicqWGPbqAcqGGNaWjcqGHGjsUcqWGPbqAcqGG8baFieWacqWFdbWqdaWgaaWcbaaccaGae4NeI0IaemyAaKgabeaakiabcYcaSiab=Hha4naaBaaaleaacqWGPbqAaeqaaOGaeiilaWIae8xta0KaeiilaWIae83uamLae8hlaWIae8xEaK3aaSbaaSqaaiabdMgaPbqabaGccqWFSaalcqWFcbGqcqGGPaqkcqGHDisTaeaacqqGGaaicqqGGaaicqqGGaaicqqGGaaicqqGGaaicqqGGaaicqqGGaaicqqGGaaicqqGGaaicqqGGaaicqqGGaaicqqGGaaicqqGGaaicqqGGaaicqqGGaaidaWcaaqaaGGaciab9f7aHbqaaiabdsfaujab+jHiTiabigdaXiabgUcaRiab9f7aHbaadaWdbaqaaiabdAgaMnaaBaaaleaacqWGobGtaeqaaOGaeiikaGIae8hEaG3aaSbaaSqaaiabdMgaPbqabaGccqGG8baFiiWacqaF8oqBdaWgaaWcbaGaemyCaehabeaakiabcYcaSGGabiab7n6atnaaBaaaleaacqWGXbqCaeqaaOGaeiykaKIaemOzay2aaSbaaSqaaiabdcfaqjabdgeabbqabaGccqGGOaakcqWF5bqEdaWgaaWcbaGaemyAaKgabeaakiabcYha8jab=jgaInaaBaaaleaacqWGXbqCaeqaaOGaeiykaKcaleqabeqdcqGHRiI8aOGaemiCaaNaeiikaGIaeWhVd02aaSbaaSqaaiabdghaXbqabaGccqGGSaalcqWEJoWudaWgaaWcbaGaemyCaehabeaakiabcYha8jab8T7aSjabcYcaSiab9r8a0jabcYcaSiab9j7aIjabcYcaSiab9z8aMjabcMcaPiabdchaWjabcIcaOiab=jgaInaaBaaaleaacqWGXbqCaeqaaOGaeiykaKIaemizaqMaeiikaGIaeWhVd02aaSbaaSqaaiabdghaXbqabaGccqGGSaalcqWEJoWudaWgaaWcbaGaemyCaehabeaakiabcYcaSiab=jgaInaaBaaaleaacqWGXbqCaeqaaOGaeiykaKcaaaa@AFAC@

Similarly, the posterior probability of placing TM *q *within the expression data context into an existing cluster of TMs *t *is

p(Lq1=t|X,Σ,a) ∝n−q1tQ−1+afN(x¯q|μt,Σtnq)
 MathType@MTEF@5@5@+=feaafiart1ev1aaatCvAUfKttLearuWrP9MDH5MBPbIqV92AaeXatLxBI9gBaebbnrfifHhDYfgasaacH8akY=wiFfYdH8Gipec8Eeeu0xXdbba9frFj0=OqFfea0dXdd9vqai=hGuQ8kuc9pgc9s8qqaq=dirpe0xb9q8qiLsFr0=vr0=vr0dc8meaabaqaciaacaGaaeqabaqabeGadaaakeaacqWGWbaCcqGGOaakcqWGmbatdaWgaaWcbaGaemyCaeNaeGymaedabeaakiabg2da9iabdsha0jabcYha8Hqadiab=HfayjabcYcaSGGabiab+n6atjabcYcaSiabdggaHjabcMcaPiabbccaGiabg2Hi1oaalaaabaGaemOBa42aaSbaaSqaaGGaaiab9jHiTiabdghaXjabigdaXiabdsha0bqabaaakeaacqWGrbqucqqFsislcqaIXaqmcqGHRaWkcqWGHbqyaaGaemOzay2aaSbaaSqaaiabd6eaobqabaGccqGGOaakcuWF4baEgaqeamaaCaaaleqabaGaemyCaehaaOGaeiiFaWhccmGaeWhVd02aaSbaaSqaaiabdsha0bqabaGccqGGSaaldaWcaaqaaiab+n6atnaaBaaaleaacqWG0baDaeqaaaGcbaGaemOBa42aaSbaaSqaaiabdghaXbqabaaaaOGaeiykaKcaaa@5EA9@ where x¯q=∑ci=qxinq
 MathType@MTEF@5@5@+=feaafiart1ev1aaatCvAUfKttLearuWrP9MDH5MBPbIqV92AaeXatLxBI9gBaebbnrfifHhDYfgasaacH8akY=wiFfYdH8Gipec8Eeeu0xXdbba9frFj0=OqFfea0dXdd9vqai=hGuQ8kuc9pgc9s8qqaq=dirpe0xb9q8qiLsFr0=vr0=vr0dc8meaabaqaciaacaGaaeqabaqabeGadaaakeaaieWacuWF4baEgaqeamaaCaaaleqabaGaemyCaehaaOGaeyypa0ZaaSaaaeaadaaeqbqaaiab=Hha4naaBaaaleaacqWGPbqAaeqaaaqaaiabdogaJnaaBaaameaacqWGPbqAaeqaaSGaeyypa0JaemyCaehabeqdcqGHris5aaGcbaGaemOBa42aaSbaaSqaaiabdghaXbqabaaaaaaa@3E6A@. and within the binding data context it is p(Lq2=t|B,a)∝n−q2tQ−1+a∏ci=qfPA( yi|bq)
 MathType@MTEF@5@5@+=feaafiart1ev1aaatCvAUfKttLearuWrP9MDH5MBPbIqV92AaeXatLxBI9gBaebbnrfifHhDYfgasaacH8akY=wiFfYdH8Gipec8Eeeu0xXdbba9frFj0=OqFfea0dXdd9vqai=hGuQ8kuc9pgc9s8qqaq=dirpe0xb9q8qiLsFr0=vr0=vr0dc8meaabaqaciaacaGaaeqabaqabeGadaaakeaacqWGWbaCcqGGOaakcqWGmbatdaWgaaWcbaGaemyCaeNaeGOmaidabeaakiabg2da9iabdsha0jabcYha8Hqadiab=jeacjabcYcaSiabdggaHjabcMcaPiabg2Hi1oaalaaabaGaemOBa42aaSbaaSqaaGGaaiab+jHiTiabdghaXjabikdaYiabdsha0bqabaaakeaacqWGrbqucqGFsislcqaIXaqmcqGHRaWkcqWGHbqyaaWaaebuaeaacqWGMbGzdaWgaaWcbaGaemiuaaLaemyqaeeabeaakiabcIcaOiabbccaGiab=Lha5naaBaaaleaatCvAUfeBSjuyZL2yd9gzLbvyNv2CaeHbuLwBLnhiov2DGi1BTfMBaGabciaa9LgaaeqaaOGaeiiFaWNae8Nyai2aaSbaaSqaaiabdghaXbqabaGccqGGPaqkaSqaaiabdogaJnaaBaaameaacqWGPbqAaeqaaSGaeyypa0JaemyCaehabeqdcqGHpis1aaaa@6998@. Posterior probabilities of placing TMs into new clusters of TMs within each context are similarly derived as for EB profile classification variables ***C*.**

All other conditional posterior distributions are similar to the simple infinite mixture models [21]. The Gibbs sampler proceeds to sample first EB profile classification variables ***C***, then local groupings of TMs within the expression and binding contexts ***C***, and then the rest of the parameters in the model. To alleviate the problem of "slow mixing", we apply heuristic annealing adjustment [18,21]. Previously, we demonstrated that such modifications preserve the topology of the posterior distribution of clusterings [18]. TMs are then formed based on the marginal posterior distributions of the classification variables **C **and **L(C)**. Summarizing the posterior distribution of **C **and **L(C) **generated by the Gibbs sampler is generally a non-trivial problem due to the label switching[64,65]. We circumvent this problem by summarizing posterior distributions of **C **and **L(C) **in terms of Posterior Pairwise Probabilities (PPPs) and Posterior Binding Probabilities (PBPs). Given the sequence of parameters (***C***^***g***^, ***L***^***g***^, ***B***^***g***^) after *B *burn in iterations, *g *= *B *+ *1*, ..., *G*, generated by the Gibbs sampler, for each pair of genes, PPP is the proportion of Gibbs samples after burn-in in which the two genes are placed in the same TM. For each gene-TF pair, PBP is the proportion of Gibbs samples after burn-in in which the specific TF is associated with the TM that contains the specific gene.

### Inferring transcription factors from PBP and binding p-value

Once we select gene clusters based on average PPP distance and proper Gene Ontology annotations we can infer associated TFs by either PBP or binding p-values. The first method transformed binding p-value to a boolean value based on the p-value cut-off threshold (0.001). Each TF was then examined to determine if it was significantly bound to the promoters of the gene cluster using a Fisher exact test (p-value <= 0.005). The second method calculated the average PBP between gene clusters and each TF. Those TFs with PBP >= 0.1 were considered significant. The selection of thresholds for significance is established empirically to balance the sensitivity and specificity of candidate TFs. This is the same cut-off threshold as used in the original publication [6]. The PBP threshold was chosen by examining the distribution of all PBPs to select the cut-off with pretty much the same level of specificity that was achieved by the p-value cut-off. Cluster size of 10 was somewhat ad-hoc cut-off aimed at getting reasonable level of statistical power to detect significant Gene Ontologies correlating with TMs.

It is important to emphasize that ROC curves presented before are completely independent of these threshold selections. These thresholds are only used when finally constructing TM's based on the posterior distribution generated by the Gibbs sampler. ROC's are designed to systematically compare true and false positive results using all possible ways to automatically construct TM's from the Gibbs sampler output.

## Abbreviations

Context Specific Infinite Mixture Model (CSIMM)

Transcription Factor (TF)

Transcriptional Module (TM)

Position Weight Matrix (PWM)

Receiver Operating Characteristic (ROC)

true positive rate (TPR)

false positive rate (FPR)

Chromatin Immuno-Precipitation on Chip (ChIP-chip)

Posterior Pairwise Probabilities (PPP)

Posterior Binding Probabilities (PBP)

Expression-ChIP Infinite Mixture (ECIM)

## Availability and requirements

We have implemented ECIM algorithm within the R package *gimmR *which can be downloaded our website .

## Authors' contributions

XL developed the statistical model, wrote appropriate computer programs, performed all analyses, interpreted results and drafted the manuscript. MM conceived the methodology and provided guidance in the development, design, analysis, interpretation of results, and drafting of the manuscript, and SS contributed to the statistical details of the method. WJJ assisted with interpretation of identified TMs and BJA provided overall guidance on biological interpretation of results.

## Supplementary Material

Additional file 1 Liu-et-al-TranscriptionalModuleDiscovery-WebSupplement4.doc. Additional data analysis results and remaining prior and posterior conditional probability distribution formulas. Click here for file

Additional file 2 SupplementalTable1_GRAM_parameters.xls. lists of GRAM's parameters and corresponding true and false positive rates. Click here for file

Additional file 3 SupplementalTable2_SAMBA_parameters.xls. lists of SAMBA's parameters and corresponding true and false positive rates. Click here for file

Additional file 4 SupplementalTable3_GRAM_ECIM_GO.xls. The comparison of TMs generated by GRAM and ECIM using the Sporulation-CellCycle expression datasets used in Figures 2. Click here for file

Additional file 5 SupplementalTable4_TMs.xls. Details of all Sporulation-CellCycle modules depicted in Figures 7 and 8B in the main text. Click here for file

Additional file 6 SupplementalTable5_TMs_Harbinson.xls. Details of all Sporulation-CellCycle modules Identified using Sporulation-CellCycle dataset and the Harbinson ChIP-chip dataset[60]. Click here for file
