# Supplementary material for: Bayesian hierarchical model for transcriptional module discovery by jointly modeling gene expression and ChIP-chip data
Source: BMC Bioinformatics. 2007 Aug 3;8:283. doi: 10.1186/1471-2105-8-283 (PMC1994961; doi:10.1186/1471-2105-8-283)
Supplement: Additional file 6 — SupplementalTable5_TMs_Harbinson.xls. Details of all Sporulation-CellCycle modules Identified using Sporulation-CellCycle dataset and the Harbinson ChIP-chip dataset[60]. [file 1471-2105-8-283-S6.doc]

**Web Supplement**

Bayesian hierarchical model for transcriptional module discovery by jointly modeling gene expression and ChIP-chip data

X. Liu^1,2^, W. J. Jessen^2^, S. Sivaganesan^3^, B. J. Aronow^2^, and M. Medvedovic^1,2*^

This document, software and additional supplemental information can be found at [**http://eh3.uc.edu/ecim**](http://eh3.uc.edu/ecim)

### OUTLINE:

1. Functional coherence of TMs identified by using the (Harbison et al. 2004) ChIP-chip data
2. Remaining prior and posterior conditional probability distributions in the contex-specific infinite mixture model

### 1. Functional coherence of TMs identified by using the (Harbison et al. 2004) ChIP-chip data

**Figure S2**: ROC curves for combined sporulation-cellCycle datasets with new ChIP-chip dataset of (Harbison et al. 2004)(203 YPD Transcription Factors)

### 2. Remaining prior and posterior conditional probability distributions in the contex-specific infinite mixture model:

**Variables in the model:**

**x**_i_=(x_i1_, x_i2_,…, x_iM_) , i=1,…,T observed gene expression profiles for all T genes

**μ**_q_=(μ_q1_,…, μ_qM_), q=1,…,Q the mean profile for global cluster q

**x**_i_^f^=$\text{(x}_{\text{i r'}_{\text{f}}+1},....,\text{x}_{\text{i r'}_{\text{f}}+\text{r}_{\text{f}}})$ where r’_f_=r_1_+…+r_f-1_, f=1,…,R is the expression profile for gene i within context f, i=1,…,Q, f=1,…,R

$\mu_{\text{tf}}^{\text{*}}$, mean expression profile for the local cluster t within context f

**M**=(**μ**_1_,…,**μ**_Q_)

**S**=(**Σ**_1_,…,**Σ**_Q_), where each **Σ**_q_ is a diagonal matrix with context-specific cluster variances on the diagonal. That is **Σ**_q_=diag($\text{(σ}_{\text{q1}}^{2},....,\text{σ}_{\text{q1}}^{2}),\text{(σ}_{\text{q2}}^{2},....,\text{σ}_{\text{q2}}^{2}),...,\text{(σ}_{\text{qR}}^{2},....,\text{σ}_{\text{qR}}^{2})$)

**M**^*^=$\text{[(}\mu_{\text{1 }1}^{\text{*}},....,\mu_{\text{K}_{1}1}^{\text{*}}),...,(\mu_{\text{1 R}}^{\text{*}},....,\mu_{\text{K}_{\text{R}}\text{R}}^{\text{*}})]$

**S**^*^=$\text{[}\Sigma_{1}^{*},...,\Sigma_{R}^{*}]$, where $\Sigma_{\text{f}}^{*}=(\text{σ}_{\text{q}}^{\text{*}})^{2}I$for f=1,…,R

Hyperparameters **τ**, **β** and **φ** are all assumed to be context-specific:

**τ=(**τ_1_,…,τ_R_), **β=(**β_1_,…, β_R_), **φ=(**φ_1_,…, φ_R_).

**The joint distribution of all variables from the model in Figure 1 of the paper:**

*p*(**X**, **C**, **L**, **M**, **M**^*^, **S,** α, a, **λ**, τ, β, φ) = *p*(**X**| **C**, **M**, **S**)*p*(**C**|α)*p*(**M**|**L**,**M**^*^)*p*(**S**|β, φ)

*p*(**L**|**C**,a)*p*(**M**^*^|**λ**, τ)*p*(α)*p*(a)*p*(**λ**)*p*(τ)*p*(β)*p*(φ)

**Conditional distributions given parent nodes:**

$p(\text{ }x_{\text{i}}\text{ }|\text{c}_{\text{i}}=\text{q, }M\text{,}S)=f_{N}(\text{ }x_{\text{i}}\text{ }|\text{ }\mu_{\text{q}}\text{,}\Sigma_{\text{q}})$.

$$p(\text{ (}\mu_{\text{q1}}\text{,}\mu_{\text{q2}}\text{,...,}\mu_{\text{qR}}\text{) }|\text{ }L\text{, }M^{*}\text{,σ}^{\text{*}})=(f_{N}(\mu_{\text{q1}}\text{ }|\text{ }\mu_{\text{L}_{\text{q1}}1}^{*}\text{,}\Sigma_{1}^{\text{*}}),f_{N}(\mu_{\text{q2}}\text{ }|\text{ }\mu_{\text{L}_{\text{q2}}2}^{*}\text{,}\Sigma_{2}^{\text{*}}),...,f_{N}(\mu_{\text{qR}}\text{ }|\text{ }\mu_{\text{L}_{\text{qR}}\text{R}}^{*}\text{,}\Sigma_{\text{R}}^{\text{*}})$$

$p(\text{ c}_{\text{i}}=\text{q }|\text{ }C_{-\text{i}}\text{, }\alpha\text{)}\propto\frac{\text{n}_{-\text{i,q}}}{\text{T-1}+\text{α}}$**,** q=1,…,Q, $p(\text{ c}_{\text{i}}\neq\text{c}_{\text{i'}}\text{, i'}\neq\text{i }|\text{ }C_{-\text{i}}\text{, }\alpha\text{)}\propto\frac{\text{α}}{\text{T-1}+\text{α}}$**,** i=1,…,T, where n_-i,q_ is the number of profiles placed in global cluster q not counting the profile i

$p(\text{L}_{\text{qf}}=\text{t}|\text{C},\text{a})\text{ }\propto\text{ }\frac{\text{n}_{\text{-qft}}}{\text{Q-1}+\text{a}}$, t=1,..,Q, $p(\text{L}_{\text{kf}}\neq\text{L}_{\text{k'f}},\forall\text{k'}\neq\text{k}|\text{C},\text{a})\text{ }\propto\frac{\text{a}}{\text{Q-1}+\text{a}}$ where n_-qft_ is the number of global clusters currently placed in local cluster t within context f without counting the q^th^ global cluster

$p(\mu_{\text{tf}}^{\text{*}}|\lambda_{\text{f}}\text{,τ}_{\text{f}})=f_{N}(\mu_{\text{tf}}^{\text{*}}|\lambda_{\text{f}},\text{τ}_{\text{f}}^{\text{-1}}I)$ $p(\text{σ}_{\text{tf}}^{\text{-2}}|\text{β}_{\text{f}}\text{,φ}_{\text{f}})=f_{G}(\text{σ}_{\text{tf}}^{\text{-2}}|\frac{\text{β}_{\text{tf}}}{2},\frac{\text{β}_{\text{f}}\text{φ}_{\text{f}}}{2})$, f=1,…,R

$$p(\text{φ}_{\text{f}}\text{|σ}_{\text{xf}}^{2})=f_{G}(\text{φ}_{\text{f}}|\frac{1}{2},\frac{\text{σ}_{\text{xf}}^{\text{-2}}}{2})$$

$$p(\text{β}_{\text{f}})=f_{G}(\text{β}_{\text{f}}|\frac{1}{2},\frac{1}{2})$$

$$p(\text{τ}_{\text{f}}\text{|σ}_{xf}^{2})=f_{G}(\text{τ}_{\text{f}}|\frac{1}{2},\frac{\text{σ}_{xf}^{2}}{2})$$

$$p(\lambda_{\text{f}}|\mu_{\text{xf}},\text{σ}_{\text{xf}}^{2})=f_{N}(\lambda_{\text{f}}|\mu_{\text{xf}},\text{σ}_{\text{xf}}^{2}I)$$

$$p(\text{α}^{\text{-1}})=f_{G}(\text{α}^{\text{-1}}|\frac{1}{2},\frac{1}{2})$$

where

$$\mu_{\text{xf}}=\frac{\sum_{\text{i}=1}^{\text{T}} x_{\text{i}}^{\text{f}}}{\text{T }}$$

$$\text{σ}_{\text{xf}}^{2}=\frac{\sum_{\text{i}=1}^{\text{T}} (x_{\text{i}}^{\text{f}}-\mu_{\text{xf}})`(x_{\text{i}}^{\text{f}}-\mu_{\text{xf}})}{\text{T r}_{\text{f}}\text{-1}}$$

**Posterior Conditional Distributions:**

, where $\bar{x}_{\text{tf}\bullet}=\frac{\sum_{\text{L}_{\text{c}_{\text{i}}\text{f}}=\text{t}} x_{\text{i}}^{\text{f}}}{\text{n}_{\text{tf}}^{\text{*}}}$ and $\text{n}_{\text{tf}}^{\text{*}}$ is the total number of expression profiles grouped in global clusters which are place in the local cluster t within the context f. Similarly, the variance for all global clusters place in the local cluster t within the context f is

$p(\text{ σ}_{\text{tf}}^{\text{-2}}\text{ }|\text{ }X,\text{ }M,\text{β}_{\text{f}}\text{,φ}_{\text{f}})\text{ }=\text{ }f_{G}(\text{ σ}_{\text{tf}}^{\text{-2}}\text{ }|\text{ }\frac{\text{r}_{\text{f}}\text{n}_{\text{tf}}^{\text{*}}+\text{β}_{\text{f}}}{2}\text{,}\frac{s_{\text{tf}}^{2}+\text{β}_{\text{f}}\text{φ}_{\text{f}}}{2})$, where $s_{\text{tf}}^{2}=\sum_{\text{L}_{\text{c}_{\text{i}}\text{f}}=\text{t}} \text{(}x_{\text{i}}^{\text{f}}-\mu_{\text{tf}}^{\text{*}})`\text{(}x_{\text{i}}^{\text{f}}-\mu_{\text{tf}}^{\text{*}})$

$$f(\text{ }\lambda_{\text{f}}\text{ }|\text{ }\mu_{\text{1f}}^{*}\text{,...,}\mu_{\text{K}_{f}\text{f}}^{*}\text{,τ}_{\text{f}})\text{ }=\text{ }f_{N}(\text{ }\lambda_{\text{f}}\text{ }|\text{ }\frac{\text{σ}_{\text{xf}}^{2}\frac{\sum_{\text{t}=1}^{\text{K}_{\text{f}}} \mu_{\text{tf}}^{*}}{\text{K}_{\text{f}}}+\frac{\text{τ}_{\text{f}}^{-1}}{\text{K}_{\text{f}}}\mu_{\text{xf}}}{\text{σ}_{\text{x}}^{2}+\frac{\tau^{\text{-1}}}{\text{Q}}}\text{,}\frac{\text{σ}_{\text{xf}}^{2}\frac{\text{τ}_{\text{f}}^{-1}}{\text{K}_{\text{f}}}}{\text{σ}_{\text{xf}}^{2}+\frac{\text{τ}_{\text{f}}^{-1}}{\text{K}_{\text{f}}}}I)$$

$$f(\text{ τ}_{\text{f}}^{-1}\text{ }|\text{ }\mu_{\text{1f}}^{\text{*}}\text{,...,}\mu_{\text{K}_{\text{f}}\text{f}}^{\text{*}},\lambda_{\text{f}})\text{ }=\text{ }f_{G}(\text{ τ}_{\text{f}}^{-1}\text{ }|\text{ }\frac{\text{r}_{\text{f}}\text{K}_{\text{f}}+1}{2}\text{,}\frac{\sum_{\text{t}=1}^{\text{K}_{\text{f}}} \text{(}\mu_{\text{tf}}^{\text{*}}\text{-}\lambda_{\text{f}}\text{)`(}\mu_{\text{tf}}^{\text{*}}\text{-}\lambda_{\text{f}}\text{)}+\text{σ}_{ \text{xf}}^{2}}{2})$$

$$f(\text{ φ}_{\text{f}}\text{ }|\text{ σ}_{\text{1f}}^{\text{-2}}\text{,...,σ}_{\text{K}_{\text{f}}\text{f}}^{\text{-2}}\text{,β}_{\text{f}})=\text{ }f_{G}(\text{φ}_{\text{f}}\text{ | }\frac{\text{K}_{\text{f}}\text{β}_{\text{f}}+1}{2}\text{ , }\frac{\text{β}_{\text{f}}\sum_{\text{t}=1}^{\text{K}_{\text{f}}} \text{σ}_{\text{tf}}^{\text{-2}}+\text{σ}_{\text{xf}}^{\text{-2}}}{2})$$

$$f(\text{ β}_{\text{t}}\text{ }|\text{ σ}_{\text{tf}}^{\text{-2}}\text{,...,σ}_{\text{tf}}^{\text{-2}}\text{,φ}_{\text{f}})\text{ }\alpha\text{ }\Gamma(\frac{\text{β}_{\text{f}}}{2})(\frac{\text{β}_{\text{f}}}{2})^{\frac{\text{(K}_{\text{f}}\text{β}_{\text{f}}\text{-3)}}{2}}\text{exp}\left(-\frac{\text{β}_{\text{f}}^{\text{-1}}\text{ }}{2}\right)\prod_{\text{t}=1}^{\text{K}_{\text{f}}} \left[\text{(φ}_{\text{f}}\text{σ}_{\text{tf}}^{\text{-2}}\text{)}^{\frac{\text{β}_{\text{f}}}{2}}\text{ exp}\left(\text{-}\frac{\text{σ}_{\text{tf}}^{\text{-2}}\text{φ}_{\text{f}}\text{β}_{\text{f}})}{2}\right)\text{ }\right]$$

$$f(\text{ α }|\text{ Q,T})\text{ }\alpha\text{ }\frac{\text{α}^{\text{Q-}\frac{3}{2}}\exp(-\frac{1}{2\text{α}})\Gamma(\text{α})}{\Gamma(\text{T}+\text{α})}$$

The posterior distributions for **C** and **L** are now:

$p(\text{ c}_{\text{i}}=\text{q }|\text{ }C_{-\text{i}}\text{, }L\text{,}x_{\text{i}}\text{,}M\text{,}\Sigma\text{)}\propto\frac{\text{n}_{-\text{i,q}}}{\text{T-1}+\text{α}}\text{ }f_{N}(\text{ }x_{\text{i}}\text{ }|\text{ }\mu_{\text{q}}\text{,}\Sigma_{\text{q}}\text{)}$

$p(\text{ c}_{\text{i}}\neq\text{c}_{\text{i'}}\text{, i'}\neq\text{i }|\text{ }C_{-\text{i}}\text{, }x_{\text{i}}\text{, }\mu_{x}\text{,σ}_{x}^{2}\text{)}\propto\frac{\text{α}}{\text{T-1}+\text{α}}\left(\left[\int f_{N}(\text{ }x_{\text{i}}\text{ }|\text{ }\mu_{\text{q}}\text{,}\Sigma_{\text{q}}\text{) }p(\mu_{\text{q}}\text{,}\Sigma_{\text{q}}|\lambda\text{,}\tau)\text{ d}\mu_{\text{q}}\text{d}\Sigma_{\text{q}}\right]\right)$

$$p(\text{L}_{\text{qf}}=\text{t}|X,\text{a})\text{ }\propto\text{ }\frac{\text{n}_{\text{-qft}}}{\text{Q-1}+\text{a}}f_{N}(\text{ }\bar{x}_{\text{f}}^{\text{q}}|\mu_{\text{tf}}^{*},\frac{\text{σ}_{\text{tf}}^{2}}{\text{n}_{\text{q}}}I\text{)}$$

,

where $\text{n}_{\text{-i,q}}$ is the number of profiles in global cluster q without counting profile i, and $\text{n}_{\text{-qft}}$ is the number of global clusters grouped into local cluster t within context f not counting q^th^ global cluster, and $\bar{x}_{\text{f}}^{\text{q}}=\frac{\sum_{\text{c}_{\text{i}}=\text{q}} x_{\text{i}}^{\text{f}}}{\text{n}_{\text{q}}}$.

Reference List

1. Harbison CT, Gordon DB, Lee TI, Rinaldi NJ, Macisaac KD, Danford TW, Hannett NM, Tagne JB, Reynolds DB, Yoo J, Jennings EG, Zeitlinger J, Pokholok DK, Kellis M, Rolfe PA, Takusagawa KT, Lander ES, Gifford DK, Fraenkel E, and Young RA, Transcriptional regulatory code of a eukaryotic genome. Nature 431: 99-104, 2004.

2. Gasch AP, Spellman PT, Kao CM, Carmel-Harel O, Eisen MB, Storz G, Botstein D, and Brown PO, Genomic expression programs in the response of yeast cells to environmental changes. Mol.Biol.Cell 11: 4241-4257, 2000.

3. Ihmels J, Bergmann S, and Barkai N, Defining transcription modules using large-scale gene expression data. Bioinformatics. 20: 1993-2003, 2004.
